# Rival male chemical cues evoke changes in male pre- and post-copulatory investment in a flour beetle

**DOI:** 10.1093/beheco/arv047

**Published:** 2015-04-29

**Authors:** Sarah M. Lane, Joanna H. Solino, Christopher Mitchell, Jonathan D. Blount, Kensuke Okada, John Hunt, Clarissa M. House

**Affiliations:** ^a^Centre for Ecology and Conservation, College of Life & Environmental Sciences, University of Exeter, Penryn Campus, Cornwall TR10 9EZ, UK,; ^b^Liverpool School of Tropical Medicine, Vector Control Department, Pembroke Place, Liverpool L3 5QA, UK,; ^c^Hawkesbury Institute for the Environment, University of Western Sydney, Hawkesbury Campus, Penrith, New South Wales 2751, Australia, and; ^d^Laboratory of Evolutionary Ecology, Graduate School of Environmental Science, Okayama University, Tsushima-naka 1-1-1, Okayama, Japan

**Keywords:** chemical cues, cuticular hydrocarbons, ejaculate expenditure, *Gnatocerus cornutus*, sperm competition risk.

## Abstract

Males can gather information on the risk and intensity of sperm competition from their social environment. Recent studies have implicated chemosensory cues, for instance cuticular hydrocarbons (CHCs) in insects, as a key source of this information. Here, using the broad-horned flour beetle (*Gnatocerus cornutus*), we investigated the importance of contact-derived rival male CHCs in informing male perception of sperm competition risk and intensity. We experimentally perfumed virgin females with male CHCs via direct intersexual contact and measured male pre- and post-copulatory investment in response to this manipulation. Using chemical analysis, we verified that this treatment engendered changes to perfumed female CHC profiles, but did not make perfumed females “smell” mated. Despite this, males responded to these chemical changes. Males increased courtship effort under low levels of perceived competition (from 1–3 rivals), but significantly decreased courtship effort as perceived competition rose (from 3–5 rivals). Furthermore, our measurement of ejaculate investment showed that males allocated significantly more sperm to perfumed females than to control females. Together, these results suggest that changes in female chemical profile elicited by contact with rival males do not provide males with information on female mating status, but rather inform males of the presence of rivals within the population and thus provide a means for males to indirectly assess the risk of sperm competition.

## Lay Summary

Males adjust both courtship effort and ejaculate expenditure when mating with females that are coated in the chemical cues of other males. Using a manipulative approach, we show that male flour beetles use the chemical cues of rival males left behind on virgin female cuticles to assess sperm competition risk. These cues do not make virgin females more chemically similar to mated females but appear to allow males to indirectly assess competition within the population.

## INTRODUCTION

Sperm competition occurs when sperm from 2 or more males compete within the female genital tract to fertilize a female’s ova (Parker 1970). There is good evidence that the relative number of sperm represented within the female is an important determinate of success in sperm competition (Wedell et al. 2002; Parker and Pizzari 2010; Kelly and Jennions 2011). However, males cannot always produce limitless supplies of sperm as sperm production can be costly (Wedell et al. 2002). Furthermore, the energetic costs associated with sperm production (Olsson et al. 1997) are expected to trade off with other aspects of reproduction such as obtaining a mate or investing in future reproductive events (Liljedal et al. 1999; Fitzpatrick et al. 2012; Parker et al. 2013). Consequently, males should adjust their ejaculate investment according to the benefit accrued from a mating and the risk (the probability that sperm from different ejaculates will compete—Parker 1970) and intensity (the number of competing ejaculates—Engqvist and Reinhold 2006) of sperm competition. Under this scenario, males should maximize ejaculate expenditure when under sperm competition risk but actually decrease ejaculate expenditure as the intensity of sperm competition increases beyond 1 competing ejaculate and the benefits of investing more diminish (Engqvist and Reinhold 2006).

A male’s ability to respond to changes in sperm competition risk and intensity is entirely dependent on his ability to gather information to assess this risk and intensity accurately (Parker et al. 1997). Males can acquire such information from a variety of cues in their socio-sexual environment [e.g., visual—presence of rival males during mating *Drosophila pseudoobscura* (Price et al. 2012), Mediterranean fruit flies *Ceratitis capitata* (Gage 1991); acoustic—male song in crickets *Teleogryllus oceanicus* (Gray and Simmons 2013); Tactile—*Drosophila melanogaster* (Bretman et al. 2011)], and recent empirical evidence illustrates that males rely on these cues, often in combination (Bretman et al. 2011; Thomas 2011). However, not all cues provide an equal breadth of information. Visual, audio, and tactile cues for instance can reliably indicate the local presence of competitors but denote nothing about a female’s mating status. Chemosensory cues on the other hand offer males a 2-fold insight into the risk and intensity of sperm competition (e.g., Carazo et al. 2004; Sirot et al. 2011; Garbaczewska et al. 2013). Males of many species use olfactory cues in the form of scent marking to communicate their presence to rival males (e.g., meadow voles; delBarco-Trillo and Ferkin 2004). Simultaneously, courtship and copulation can elicit changes in a female’s chemical profile, changes that can be triggered by the transfer of male-derived chemicals (Siva-Jothy and Stutt 2002; Andersson et al. 2004; Wedell 2005) or through physiological mechanisms within the female herself (Scott and Jackson 1990; Foster 1993; Karube and Kobayashi 1999). Therefore, unlike the aforementioned cues, chemical cues facilitate both the detection of competitors and the assessment of female mating status.

In insects, cuticular hydrocarbons (CHCs), semiochemicals transferred directly from male to female via contact, have been shown to elicit behavioral responses to sperm competition risk. In the fruit fly, *D. melanogaster*, experimentally perfuming virgin females with the CHCs of mated females induced males to mate for longer (Scott 1986; Friberg 2006). Furthermore, male Australian field crickets (*Teleogryllus oceanicus*) have been shown to distinguish between the individual profiles of rival males left behind on the female cuticle in order to detect both the risk and intensity of sperm competition (Thomas and Simmons 2009). These studies implicate the importance of CHCs as cues of female mating status and therefore the risk and intensity of sperm competition. However, research to investigate how CHCs may select on male reproductive traits is limited to species in the genus *Drosophila* and *Teleogryllus*. Therefore, further studies across a wider number of taxa are required to determine whether male responsiveness to contact-derived CHCs is a general phenomenon that will drive the evolution of male sexual traits.

Females of the broad-horned flour beetle, *Gnatocerus cornutus*, exhibit moderate levels of polyandry and repeated mating in populations maintained at an equal sex ratio (Clarissa M. House, unpublished data). Highly aggressive males limit the access of loser males to females through male–male competition, repeated mating with the same female and extended periods of post-copulatory mate guarding (Clarissa M. House, unpublished data). Previous studies have shown that males who lose fights become less aggressive and increase their investment in ejaculates for 4 days after a fight (Okada et al. 2010), a response to relative social competitiveness that has also been shown in birds (Pizzari et al. 2007). This response indicates that males of this species respond to sperm competition risk, but it is unknown whether males can perceive the risk and intensity of sperm competition in the physical absence of local competitors. *G. cornutus* exhibit a highly tactile form of courtship, in which the male mounts the female and stimulates her, drumming his tibia along her abdomen until she allows him to mate with her. Such tactile courtship can last for more than 10min (Sarah M. Lane, personal observation), which may provide an opportunity for the exchange of CHCs. Thus, there is the potential for contact-derived semiochemicals to elicit changes in the female chemical profile and provide information on the risk and intensity of sperm competition in *G. cornutus.*

Here, we manipulated the chemical profile of virgin females by facilitating contact between males and females to investigate whether contact-derived male CHCs retained on female cuticles influence pre-copulatory (courtship effort) and post-copulatory (ejaculate expenditure) male investment. First, we tested the hypotheses that males can assess sperm competition risk and intensity from chemical cues of female mating status and invest most in courtship when the risk of sperm competition is high and least with increased intensity of sperm competition. Next, we tested the hypothesis that males respond to the risk of sperm competition perceived via these chemical cues by allocating more sperm to an ejaculate. Finally, using gas chromatography-mass spectrometry (GC-MS), we tested whether perfuming virgin females with the CHCs of males changed their chemical profiles such that these females were more chemically similar to mated females and thus whether rival male CHCs provide a cue of female mating status.

## MATERIALS AND METHODS

### Stock populations and rearing protocols

*Gnatocerus cornutus* are a stored product pest that feed on a variety of grains, flours, yeasts, and dry animal products (Linsley 1944; Zakladnoi and Ratanova 1987), so replicating their natural environment is easy. Beetles used in this study were taken from stock populations of *G. cornutus* derived from the Japanese National Food Research Institute (NFRI), at which beetle cultures have been maintained for more than 50 years (see Okada et al. 2006 for details of origin and culture conditions). In our laboratory in the United Kingdom, we replicated these culture conditions closely. In brief, mixed sex populations have been maintained since 2012 in pots (Thermoscientific Nalgene 500mL, 120mm o.d.[outer diameter]) containing 50 individuals. These stock populations are reared on wholemeal flour enriched with 5% yeast and incubated at 27 °C with 60% humidity on a 14:10h light:dark lighting cycle (Okada et al. 2006). Every 3–4 weeks, a random selection of final instar larvae is removed from each stock pot (*n* = 18) and placed into six 24-well plates as pupation is inhibited at moderate to high larval density (Tsuda and Yoshida 1985). At eclosion, 25 male and 25 female adults are randomly selected to form the parents of the next generation.

### Preliminary investigations

During our preliminary investigations, we conducted 2-hr observations of small, equal sex-ratio populations (number of populations = 59; *n* = 4♀ and 4♂ per population; total *N* = 236♀ and 236♂) of uniquely marked males and females that were held in close proximity (mating/fighting arenas). We recorded the number of mates acquired by females and males and the number of male–male agonistic contests. Average female mating success (i.e., mating with different males) was 1.01 with a variance of 1.68 compared with males whose average mating success was 1.21 with a variance of 2.51 (Clarissa M. House, unpublished data; calculated according to Shuster and Wade 2003). During this time period, males repeatedly mated with the same female up to 8 times (mean = 2.73), which is likely to dilute or displace rival males sperm. Models of sperm competition integrate the patterns of male sperm precedence and the probability that a female will re-mate with another male (Engqvist and Reinhold 2006). However, in this system, a female will engage in polyandry as well as repeated mating with the same male, which should influence the numerical representation of rival sperm in the female sperm storage organs. Thus, it is unclear when male *G. cornutus* should perceive a risk of sperm competition after a female has mated 1 or more times.

### Experimental animals

We collected final instar larvae from lab stocks daily and placed them into 24-well plates until eclosion. The day after eclosion, we transferred adults into single sex 24-well plates to prevent interactions between conspecifics. The lids of the male-only 24-well plates were secured with masking tape to prevent tactile and visual contact between males that have previously been shown to influence investment in ejaculates (Okada et al. 2010). All adults were provided with ad libitum wholemeal flour and maintained as described above.

### Experiment 1: pre-copulatory investment

To determine the potential for males to detect cues about the risk and intensity of sperm competition from CHCs transferred from males to females via contact, we perfumed 17-day old virgin females by vortexing them either alone (control), or with 1, 3, or 5 virgin males. Females were placed into Eppendorf tubes (1.5mL) containing the males and vortexed for 30 secs on a low setting, facilitating contact and CHC transfer between the sexes while preventing courtship and copulation. The males used during vortexing were discarded immediately after and were not used in subsequent mating trials. Thirty minutes after vortexing, we paired the vortexed females with random virgin males of the same age and recorded the number of times the males courted with them during a 40 min observation period. These observations continued for the whole 40min, even if mating occurred, as a male will continue to court the same female even after he has successfully mated with her (Clarissa M. House, personal observation).

#### Statistical analyses

To analyse the effect of perfuming treatment on male courtship effort, we conducted a generalized linear model (GLM). Because courtship effort was not normally distributed and highly overdispersed, we used a quasi-Poisson error family in our model that allowed us to account for this overdispersion. To further investigate the effect of treatment, we conducted multiple post hoc comparisons between the 4 treatments (control, 1 male, 3 males, and 5 males) using a Tukey’s Honestly Significant Difference (HSD) test.

### Experiment 2: post-copulatory investment and re-mating rate

#### Perfuming and mating trials

To investigate the effect of the presence of male-derived CHCs on ejaculate investment, we assigned virgin females to 1 of 3 treatments—control, sham, or perfumed—6 days after eclosion (as above). On Day 17, perfumed females were individually placed into Eppendorf tubes (1.5mL) containing 3 random virgin males of the same age. These beetles were then vortexed for 30 secs, 30min before mating (as described above) and separated immediately afterward. Once again, males used as a source of chemical cues were not used for the mating trials. Sham females were used to investigate the effects of vortexing per se on mating behavior and were vortexed alone for 30 secs.

Previous studies have shown that virgin males produce significantly larger ejaculates than mated males (Svärd and Wiklund 1986; also see Torres-Vila and Jennions 2005 for a review) and so to eradicate first-mating effects on ejaculate size and content, all males in our study were singly mated to a random nonfocal virgin female 20min prior to their focal mating (after which time, males were receptive to re-mating—Sarah M. Lane, personal observation). Females used in this first mating were frozen and discarded. For their focal mating, we paired males with a female from 1 of the 3 treatments outlined above. To control for any potential effects of female quality on ejaculate allocation, female age was standardized across all matings and all females were randomly allocated to males. We held pairs in a mating arena and observed them for 45min. Focal pairs who failed to mate were discarded from the experiment. We continued to conduct mating trials until we had obtained 40 successful focal matings for each of our treatments. Unsuccessful matings were recorded and later analyzed to examine the effect of treatment on re-mating rate. If copulation occurred, females from this second mating were removed and kept individually at 27 °C for 4h. This allowed adequate time for the sperm to travel up the reproductive tract to the spermatheca (Sarah M. Lane, personal observation), before the experimental females were frozen at −20 °C. If a pair failed to mate within 45min, they were removed from the trial and discarded from the experiment. Twice-mated males were frozen for subsequent body measurement, whereas males who failed to re-mate were discarded from the experiment. We captured digital images of the dorsal view of the males’ bodies using a Leica M125 microscope with mounted camera (Leica DFC295, Leica microsystems Ltd. CH-9435 Heerbrugg) that conveyed images to a PC. We measured the width of the pronotum (to the nearest 0.01mm) as an index of body size (Okada et al. 2006) using Image J (version 1.46r). We measured each pronotum twice to calculate the repeatability of this measure based on the variance components derived from an analysis of variance (Lessells and Boag 1987), showing high repeatability (*F*_24,25_ = 120.33, *r* = 0.992±0.0034, *P* < 0.001).

#### Measuring ejaculate investment

Twenty-four hours after being frozen, females were removed from the freezer for dissection. We placed each female directly onto a fresh microscope slide, abdomen facing upward. Using 2 pairs of fine watchmaker’s forceps, we gently squeezed the female’s abdomen and carefully grasped and pulled out the reproductive tract. Removing all other tissue from the slide, we carefully separated the spermatheca from the surrounding reproductive tissue. We added 10 µL of deionized water to the center of the slide (away from the dissection area to avoid contamination of the sample), crushed the spermatheca between the forceps and placed it directly into the droplet. We stirred the sample to prevent the sperm from clumping and drew a circle around the drop to aid identification of the area under high magnification. After leaving the sample to air-dry fully, we recorded total sperm count using an Olympus BX61 microscope (Olympus Corporation, Tokyo, Japan) under phase contrast at 20× magnification. *G. cornutus* produce relatively small ejaculates of <2000 sperm making full counts possible. We thus performed sperm counts manually and the repeatability of counts of the same ejaculate was measured as described above, showing high repeatability (*F*_6,7_ = 652.464, *r* = 0.997±0.0012, *P* < 0.001). All sperm counts were performed blind by the same person throughout.

#### Statistical analyses

To analyse the effect of treatment on re-mating rate, we conducted a GLM with a binomial error family, giving individual males a binary score of either 1 or 0 to represent their success or failure to re-mate, respectively. Body size data and re-mating data for sham females were not available for this analysis and thus were not included. All statistical analyses were carried out using R (version 2.12.0).

To analyse the effect of treatment and vortexing per se on the total number of sperm transferred, we first removed all females to which no sperm had been transferred, classing these as unsuccessful matings. We then conducted a GLM on the remaining data from all 3 treatment groups. As sperm number was not normally distributed and highly overdispersed, we used a quasi-Poisson error family that allowed us to account for this overdispersion in our model. Next, we conducted a separate analysis to control for the potentially confounding effect of body size on sperm number, including pronotum width as a covariate and examined the interactions between body size and treatment. We were unable to include our sham group in this analysis as we did not have body size data for this group of females.

### Experiment 3: GC-MS analysis of experimental male and female CHC extracts

To investigate the effects of our experimental perfuming treatment on female CHC profile, we analyzed the CHC profiles of an additional subset of control and perfumed virgin females (generated as above, i.e., perfumed with 3 males but not used in the behavioral assays) along with a set of mated females, virgin males, and mated males using gas chromatography-mass spectrometry (GC-MS). All beetles used for GC-MS analysis were stored at −20 °C for 2 months before the commencement of CHC extraction. First, we randomized samples prior to the CHC extraction process to avoid any bias caused by column degradation during GC-MS. Next, we extracted CHCs from individuals by full-body immersion in 50 µL of high performance liquid chromatography (HPLC)-grade hexane with 10-ppm pentadecane as an internal standard. Individual beetles were left to soak for 5min and during the last minute, each sample was vortexed to maximize CHC extraction. After 5min, we removed the beetle from the vial using metal forceps that we cleaned in pure hexane between each sample to avoid contamination. Two microliters of the extracted CHC sample was injected into a GC-MS (Agilent 7890A gas chromatograph coupled with an Agilent 5975B mass spectrometer and an Agilent CTC PAL autosampler chilled to 5 °C, Agilent Technologies, Cheshire, UK) fitted with a DB1-MS column (30-m × 0.25-mm ID × 0.25-μm film thickness) using helium as the carrier gas. The inlet and MS transfer line were set at 250 °C and 300 °C, respectively, and the injection was run in the pulsed splitless mode. The GC oven temperature profile started at 100 °C for 1min, ramping at 20 °C/min to 250 °C, then 5 °C/min to 320 °C. GC extraction methods were uniquely designed to optimize chemical separation for *G. cornutus* on the instrument in use, and thus, the methods described here were uniquely designed for this study. Peaks were quantified using MSD Chemstation software (Agilent Technologies, version E.02.00.493), using ion 57 as the target ion to quantify the abundance of each CHC compound. Methyl-branched alkanes were identified by their mass spectra (Nelson et al. 1972), and the identities of the peaks were confirmed using retention indices (Francis and Veland 1981) that were calculated by running a straight-chain alkane standard that contained all alkanes from C_7_ to C_40_. The positions of double bonds in unsaturated hydrocarbons were determined by interpreting the mass spectra of the dimethyl disulphide derivatives. In brief, treating unsaturated hydrocarbons with dimethyl disulphide removes C=C bonds, creating a weak point in the molecule that is cleaved to produce 2 characteristic fragments. The size of these fragments can then be used to determine the position of the double bond and thus identify the compound (see Nelson et al. 1972; Buser et al. 1983 for more details).

#### Statistical analyses

GC-MS analysis identified 24 individual CHC peaks, producing quantitative data on all 24 compounds. To calculate the concentration of each compound, the area of each peak was divided by the area of the internal standard peak (peak 1) and the resulting data were log10 transformed. This allowed us to look at the variation between individual beetle’s CHC profiles as variation in this species is quantitative not qualitative (i.e., all individuals possess the 24 identified CHC compounds but in varying amounts).We then used discriminate function analyses (DFA) in order to obtain a reduced number of functions, which capture and describe the between-group variation in CHC profiles. We conducted 2 separate DFA in order to test 2 separate predictions 1) CHC profiles of perfumed females were chemically similar to those of mated females and 2) CHC profiles of perfumed females were more chemically similar to virgin males (with whom they were perfumed) than mated males. All data analysis was conducted using SPSS (version 20).

## RESULTS

### Experiment 1: pre-copulatory investment

**Figure 1:**
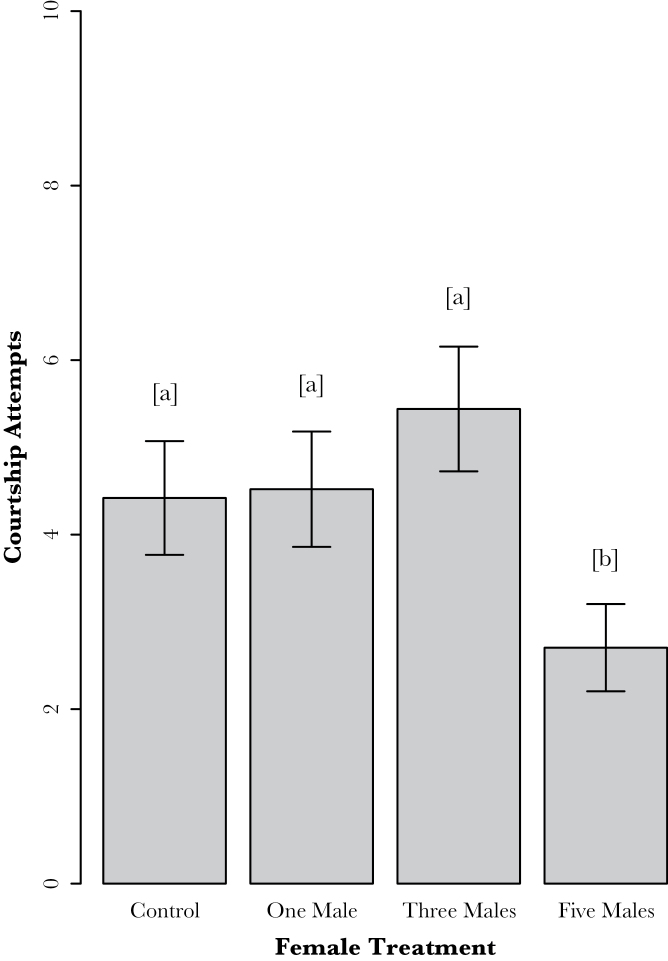
Mean (±SE) number of courtship attempts by males to females of each treatment group in Experiment 1. Different letters indicate a significant difference, males courted significantly less with females perfumed with 5 males compared with females perfumed with 3 males (*P* = 0.025).

Our analyses showed that contact-derived male CHCs retained on female cuticles had a significant effect on courtship effort (*F*_3,115_ = 3.096, *P* = 0.03). Multiple post hoc comparisons revealed that males courted females who had been vortexed with 5 males significantly less than females who had been vortexed with 3 males (*P* = 0.025; see Figure 1). Despite an increase in courtship effort between the control, 1 male, and 3 male perfuming treatments, the difference was nonsignificant between these groups (Figure 1). Nonetheless, males were, on average, most responsive to females who had been vortexed with 3 males and consequently, we used this perfuming treatment in our second experiment.

### Experiment 2: post-copulatory investment and re-mating rate

Treatment had a significant effect on re-mating rate (χ^2^ = 5.48, *P* = 0.019). Fourty-two percent of males failed to re-mate in the perfumed group compared with 22% in the control group. This result suggests that either males less readily mated with perfumed females or perfumed females less readily allowed males to mate with them, but as we did not measure courtship effort in this second experiment, we are unable to determine which.

**Figure 2:**
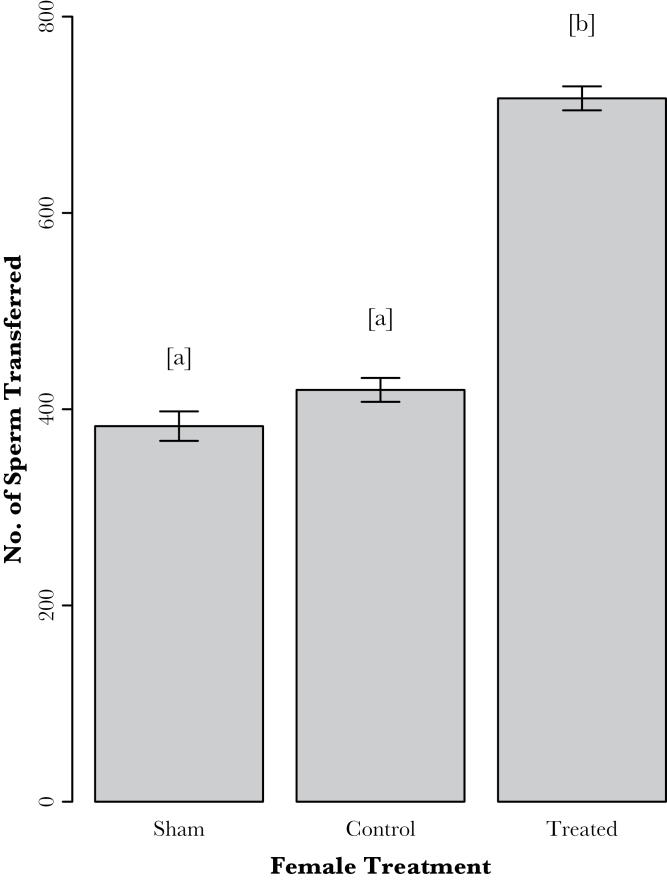
Mean (±SE) number of sperm transferred by males to females of each treatment group in Experiment 2. Different letters indicate a significant difference, males transferred significantly more sperm to females in the perfumed treatment (*P* < 0.001).

The number of sperm transferred to females differed significantly across the treatments (*F*_2,92_ = 5.86, *P* = 0.004). Post hoc analyses showed that males transferred significantly more sperm to perfumed females than to sham and control females (*F*_1,93_ = 11.64, *P* = 0.00096) (Figure 2). There was no significant difference in the number of sperm transferred to control females and sham females (*F*_1,93_ = 0.14, *P* = 0.71). In our control and perfumed females, there was no significant interaction between body size (measured here as pronotum width) and treatment (*F*_1,67_ = 2.62, *P* = 0.11). However, body size had a significant effect on the number of sperm transferred (*F*_1,68_ = 5.05, *P* = 0.03), with larger males transferring more sperm.

### Experiment 3: GC-MS analyses of experimental male and female CHC extracts

Male and female hydrocarbon profiles were composed of a mixture of straight-chained alkanes, mono- and di-methyl alkanes, and alkenes ranging from 25 to 33 hydrocarbons in length (see supplementary material for more details). Our first DFA examined the variation in the CHC profiles of our 3 groups of females—control females, perfumed females, and mated females—and produced 2 functions that together explained 100% of the between-group variation in CHCs. Estimates based on generalized cross-validation values showed that the predictive model correctly classified groups with 70.6% success.

**Figure 3:**
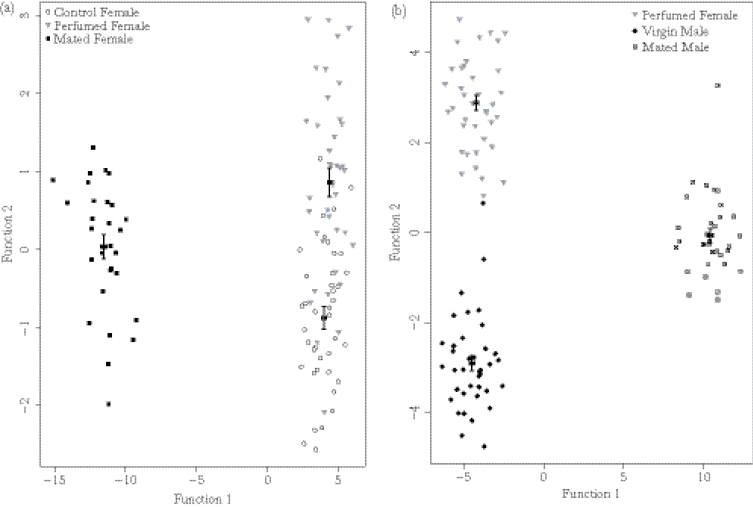
(a) Combined-groups plot showing Functions 1 and 2 derived from the discriminant function analysis of control, perfumed, and mated females. Function 1 explains 98.9% of between-group variance, separating mated females, and both groups of virgin females. Function 2 explains 1.1% of the variance, discriminating control females from perfumed and mated females. Centroids represent the averages and standard errors of each treatment. (b) Combined-groups plot showing Functions 1 and 2 derived from the discriminant function analysis of perfumed females, virgin males, and mated males. Function 1 explains 88.4% of between-group variance, separating perfumed females, and virgin males from mated males. Function 2 explains 11.6% of the variance, discriminating perfumed females from both groups of males. Centroids represent the averages and standard errors of each treatment.

Function 1 explained 98.9% of the variance in CHCs (canonical *r* ^2^ = 0.98), discriminating mated females from both control and perfumed females (Figure 3a and Table 1). Examination of the factor loadings for each of the 24 CHC peaks indicated that this discrimination was due to the amount of pentacosane, 11-methylpentacosane, and 11-methylhexacosane (peaks 2, 3, and 6, respectively). Loading factors of 0.25 or higher were interpreted as significant (Tabachnick and Fidell 1989). Function 2 described a further 1.1% of the variance in CHCs (canonical *r* ^2^ = 0.36), distinguishing control females from perfumed and mated females. Examination of the factor loadings showed that Function 2 was positively loaded to nonacosane, 3-methylnonacosane, and 3-methylhentriacontane (peaks 14, 17, and 22, respectively) while also being negatively loaded to 5-hexacosane and 13-methylnonacosane (peaks 7 and 15, respectively) (Table 1). This analysis indicates that perfumed females separate slightly from our control group but overall, the CHC profiles of mated females are very different from those of the control and perfumed females. Thus, although our perfuming treatment altered the CHC profile of perfumed females, it did not make them more chemically similar to mated females than control females.

**Table 1:** Unstandardized canonical discriminant functions evaluated at group centroids, which represent the averages. Values of constrasting signs (+/-) are highlighted to show which treatments are distinguished between at each function

Discriminant analysis 1		
Treatment	Function	
	1	2
Control females	3.983	**−0.881**
Perfumed females	4.391	0.858
Mated females	**−11.551**	0.031
Discriminant analysis 2		
Treatment	Function	
	1	2
Perfumed females	−4.207	**2.886**
Virgin males	−4.478	−2.906
Mated males	**10.392**	−0.064

Our second DFA examined the variation in CHC profiles between perfumed females, virgin males, and mated males. This DFA produced 2 functions that together described 100% of the between group variation in CHCs. Estimates based on generalized cross-validation values show that the predictive model correctly classified groups with 97.3% success. Function 1 explained 88.4% of the variance in CHCs (canonical *r* ^2^ = 0.98), discriminating both virgin groups (perfumed females and virgin males) from mated males (Figure 3b and Table 1). This separation was predominantly due to pentacosane (peak 2) to which Function 1 was positively loaded. Function 2 explained 11.6% of the variance in CHCs and separated perfumed females from both virgin and mated males (canonical *r* ^2^ = 0.86). Examination of the factor loadings showed that this discrimination was due to amounts of 5-hexacosane (peak 7) that was negatively loaded to Function 2.

## DISCUSSION

Our findings indicate that male *G. cornutus* are able to detect the local risk and intensity of sperm competition from chemical cues transferred between males and females during contact, as well as through physical interactions with rival males as has been shown previously (Okada et al. 2010). We found that we were able to experimentally alter the CHC profile of virgin females through direct intersexual contact that mimicked the tactile courtship of this species and altered the relative abundance of several hydrocarbons. In accordance with our predictions, we found that males initially increased courtship effort when under risk of competition but decreased their investment significantly as the number of rivals rose above 3, suggesting that males are sensitive to cues of both sperm competition risk and intensity. During post-copulatory investment, males responded to perfuming by significantly increasing their ejaculate expenditure when mating with perfumed females in comparison with control females, even though the chemical profile of perfumed females was not more chemically similar to mated females. An increase in ejaculate expenditure should increase a male’s probability of achieving fertilisation (Parker 1990; Parker et al. 1997); however, more work is needed to demonstrate that this increase in sperm number in *G. cornutus* is adaptive. Furthermore, it is important to note that our measure of ejaculate investment in this study (sperm counts from the spermatheca) as in other studies (Okada et al. 2010; Yamane et al. 2010) is only a proxy of ejaculate investment. Sperm utilization and storage can also be affected by female-driven factors (i.e., *Tribolium casteneum*; Edvardsson and Arnqvist 2000 and field crickets; Bretman et al. 2009) and while we are not aware of any such factors in *G. cornutus*, it is possible that our measure of ejaculate expenditure is reflective not just of patterns of male sperm allocation but female sperm utilisation also.

Contrary to our initial prediction, comparison of the CHC profiles of females perfumed with 3 rival males, control females, and mated females revealed that perfuming did not make females “smell” mated, and therefore, it is clear that our experimental males were not responding to cues about females mating status. Instead, these results raise an interesting possibility that male-derived CHCs retained on the female cuticle may provide information about the presence and density of rival males within the population and thus offer males a way to indirectly assess sperm competition risk and intensity. Specifically, our data suggest that males adjust their pre- and post-copulatory reproductive investment (i.e., courtship effort and ejaculate investment) in response to the risk and intensity of sperm competition that is detected from either the overall concentration of CHCs or the number of males’ CHCs present—we cannot say which with certainty. These results mirror evidence from Weir et al.’s 2011 meta-analysis in which an increase in operational sex ratio bias lead to a decrease in male courtship rate and aggression but an increase in copulation duration and mate guarding, further supporting the idea that males may be able to assess rival density using these chemical cues. Our results are also similar to previous studies that have directly illustrated male use of chemical cues to assess female mating status (Carazo et al. 2004; Friberg 2006) and sperm competition risk (Friberg 2006; Carazo et al. 2007; Thomas and Simmons 2009; Garbaczewska et al. 2013). However, to our knowledge, this is the first study to explicitly show that the presence of rival male chemical cues present on the cuticle of virgin females can elicit a behavioral response in males even though these cues do not make virgin females “smell” mated.

A general prediction from sperm competition models is that males are expected to allocate more sperm when mating with a virgin (Engqvist and Reinhold 2006) or in the presence of a single competitor, which is well supported empirically (Kelly and Jennions 2011). Our results do not conform exactly to these sperm competition models as by virtue of our experimental design, males responded to the chemical cues of 3 rival males not 1 as these models simulate. However, the results of Experiment 1 indicate that male *G. cornutus* do not perceive a risk of sperm competition in the presence of a single competitor. In this species, it is possible that males lack the sensory apparatus to detect the chemical signature of a single rival or perhaps the tendency of males to repeatedly mate with the same female is sufficient to dilute or displace the sperm of a single rival male and therefore a single competing ejaculate does not constitute a “risk.” Male field crickets have been shown to be able to detect the exact number of different male CHC profiles present on a female and to adjust their ejaculate in response (Thomas and Simmons 2009). Here, the results of Experiment 1 suggest that *G. cornutus* males are similarly sensitive to either the overall concentration of CHCs or the number of distinct male profiles present, but further investigation is needed. If males of this species are indeed able to distinguish between individual male profiles, this should select on sensory organs that detect unique CHCs and plasticity in ejaculate expenditure, especially if males are able to gain information about the age and quality of rivals from their CHCs alone.

Despite their potential importance, the role of chemical cues in shaping male perception of sperm competition risk is unknown, with the notable exceptions of studies in *Drosophila* (Friberg 2006) and a field cricket (Thomas and Simmons 2009). The aforementioned studies implicate (Friberg 2006) or have shown (Thomas and Simmons 2009) the importance of CHCs as a key source of sociosexual information for male sperm competition assessment, consistent with our findings. More generally, there is a growing evidence that CHCs transferred via contact are a key source of sociosexual information for both sexes. Empirical studies of *Nauphoeta cinerea* (Harris and Moore 2005), *D. melanogaster* (Scott 1986; Scott et al. 1988) and *Gryllodes sigillatus* (Weddle et al. 2013) indicate that males and females use contact-derived CHCs transferred during socio-sexual interactions to inform their mating choices. For example, female *N. cinerea* preferentially mate with males who bear the epicuticular rubbing of a single female over those who bear the rubbings of multiple females (Harris and Moore 2005), appearing to use this information to avoid mating with sperm-depleted males. Behavioral assays in *D. melanogaster* show that sex-specific CHCs transferred during mating in this species, act as antiaphrodisiacs when present on the reciprocal sex. These antiaphrodisiacs confer potential fitness benefits by reducing the chances of mating with an already mated female or a sperm-depleted male (Scott 1986; Scott et al. 1988). Finally, female *G. sigillatus* actively avoid mating with males that bear their own CHC profile, facilitating the avoidance of mating with a previous mate (Weddle et al. 2013).

The detection of rival male’s CHCs prior to mating is likely to have important consequences for the evolution of traits used during sperm competition. Whenever the environment provided by 1 individual influences the phenotype of another and variation in this environment reflects (at least in part) genetic differences between individuals, then indirect genetic effects (IGEs) will exist and the environment will be heritable (Wolf et al. 1998). In theory, IGEs can have a number of widespread implications for the evolution of phenotypic traits, including biasing the rate and direction of evolutionary change, generating evolutionary time lags in the response to selection and enabling phenotypic traits to evolve in the complete absence (or reduced levels) of additive genetic variance (Wolf et al. 1998). Moreover, IGEs may also play a central role in the maintenance of genetic variance in traits subject to strong selection (Miller and Moore 2007). It is possible that the CHCs transferred to females by rival males during mating may represent an IGE and hold important implications for the evolution of ejaculate characteristics in *G. cornutus*. Although we currently do not know the genetic basis of male CHCs in *G. cornutus*, CHCs are known to be heritable in a variety of other terrestrial arthropods (e.g., Hine et al. 2004; Thomas and Simmons 2008; Ingleby et al. 2013; Weddle et al. 2013) including beetles (Yezerski et al. 2004) and our current study shows that males are able to adjust the number of sperm in their ejaculates in response to the CHCs transferred at mating by rival males. What we do not know, however, is whether this male response varies with the genotype of rival males. Work on the field cricket (*Teleogryllus oceanicus*) suggests that IGEs between competing males engaging in sperm competition is indeed possible and can have important consequences for male reproductive success (Garcia-Gonzalez and Simmons 2007). More work is needed, however, to demonstrate the existence of IGEs in *G. cornutus* and this is the focus of our current research.

To adjust their ejaculate expenditure in response to the risk and intensity of sperm competition, males must gather information to accurately assess these states (Parker et al. 1997, 2013). Our research demonstrates that males are able to indirectly assess sperm competition risk and intensity from rival male CHCs derived from contact and retained on the female cuticle. We show that these chemical cues do not provide males with information about female mating status, but rather appear to equip males with information on the presence and perhaps density of rivals within their mating environment, and this information alone elicits an increase in reproductive investment.

## SUPPLEMENTARY MATERIAL

Supplementary material can be found at http://www.beheco.oxfordjournals.org/

## FUNDING

S.M.L. was funded by a Natural Environment Research Council (NERC) studentship, J.H.S. was funded by NERC, a Royal Society Fellowship, and a Royal Society Equipment Grant (UF120087), and C.M.H. by a Leverhulme Early Career Fellowship (ECF/2010/0067).

## Supplementary Material

Supplementary Data
