# Supplementary material for: Rival male chemical cues evoke changes in male pre- and post-copulatory investment in a flour beetle
Source: Behav Ecol. 2015 Apr 29;26(4):1021–9. doi: 10.1093/beheco/arv047 (PMC4495758; doi:10.1093/beheco/arv047)
Supplement: Supplementary Data [file supp_26_4_1021__index.html]

Rival male chemical cues evoke changes in male pre- and post-copulatory investment in a flour beetle — Rival male chemical cues evoke changes in male pre- and post-copulatory investment in a flour beetle — Rival male chemical cues evoke changes in male pre- and post-copulatory investment in a flour beetle — Supplementary Data 

# Rival male chemical cues evoke changes in male pre- and post-copulatory investment in a flour beetle

## Supplementary Data

Data files

**Files in this Data Supplement:**

- Supplementary Data - Supplementary Data
