# Supplementary material for: Rival male chemical cues evoke changes in male pre- and post-copulatory investment in a flour beetle
Source: Behav Ecol. 2015 Apr 29;26(4):1021–9. doi: 10.1093/beheco/arv047 (PMC4495758; doi:10.1093/beheco/arv047)
Supplement: Supplementary Data [file supp_arv047_Supplementary_Material_RESUB.docx]

**Supplementary Material**

**Figure 3.** A typical GC profile obtained from solvent extracts of the cuticle of male *Gnatoceruscornutus*. The *x-*axis shows the retention time (in minutes) and the *y-* axis shows the GC signal strength (in picoamperes). We found 24 unique CHC peaks in addition to the internal standard (pentadecane, peak not shown), which are characterised according to their mass spectra in Table 1.

**Figure 3.**

**Table 2.**Chemical characterization of male CHCs in *Gnatoceruscornutus*. KRI: Kovats Retention Index for each chemical compound, DMDS: diagnostic ions used for compound identification after derivation with dimethyl disulphide.

**Table 2**

| **Peak** | **KRI** | **Compound** | **Diagnostic ions** | |
| --- | --- | --- | --- | --- |
| 2 | 2495 | C_25_ | 352 | |
| 3 | 2531 | 11-MeC_25_ | 168, 227, 351 | |
| 4 | 2568 | 3-MeC_25_ | 337, 57, 351 | |
| 5 | 2594 | C_26_ | 366 | |
| 6 | 2629 | 11-MeC_26_ | 168, 238, 365 | |
| 7 | 2661 | 5-C_26_-ene | DMDS: 458, 117, 341 | |
| 8 | 2693 | C_27_ | 380 | |
| 9 | 2728 | 11-MeC_27_ | 168, 252 | |
| 10 | 2748 | Unknown |  | |
| 11 | 2759 | 11,15-diMeC_27_ | 267, 168, 197, 239 | |
| 12 | 2769 | 3-MeC_27_ | 365, 57 | |
| 13 | 2794 | C_28_ | 394 | |
| 14 | 2894 | C_29_ | 408 | |
| 15 | 2927 | 13-MeC_29_ | 252, 196 | |
| 16 | 2956 | 11,15-diMeC_29_ | 295, 168, 224, 239 | |
| 17 | 2969 | 3-MeC_29_ | 57, 393 | |
| 18 | 2993 | C_30_ | 422 | |
| 19 | 3093 | C_31_ | 436 | |
| 20 | 3126 | 15-MeC_31_ | 224, 252 | |
| 21 | 3152 | 3,19, 3,17-diMeC_31_ | 196, 224, 267, 295, 435 | |
| 22 | 3169 | 3-MeC_31_ | 57, 421 | |
| 23 | 3250 | 4,12-diMeC_31_ | 435, 71, 309, 197 | |
| 24 | 3325 | 11-MeC_33_ | 169, 337, 225, 281 | |
| 25 | 3349 | 15,17-diMeC_33_ | 295, 225, 253, 267 | |
|  | | | |  |

**Table 3.** Summary of the functions obtained from the discriminant analysis and the factor loadings of each of the 24 CHC peaks identified. Loading values of >0.3 were interpreted as significant and are highlighted in bold.

**Table 3**

| **Peak** | **Compound I.D.** | **Function 1** | **Function 2** |
| --- | --- | --- | --- |
|  | **Eigenvalue** | **8.695** | **0.433** |
|  | **% Variation** | **95.3** | **4.7** |
| **2** | C_25_ | 0.024 | 0.247 |
| **3** | 11-MeC_25_ | -0.067 | -0.032 |
| **4** | 3-MeC_25_ | 0.086 | 0.111 |
| **5** | C_26_ | -0.14 | 0.112 |
| **6** | 11-MeC_26_ | 0.141 | -0.09 |
| **7** | 5-C_26_-ene | **0.362** | 0.257 |
| **8** | C_27_ | 0.055 | -0.027 |
| **9** | 11-MeC_27_ | -0.05 | 0.195 |
| **10** | Unknown | 0.046 | 0.001 |
| **11** | 11,15-diMeC_27_ | -0.077 | 0.117 |
| **12** | 3-MeC_27_ | 0.015 | -0.011 |
| **13** | C_28_ | -0.059 | -0.036 |
| **14** | C_29_ | -0.139 | -0.197 |
| **15** | 13-MeC_29_ | -0.065 | **0.345** |
| **16** | 11,15-diMeC_29_ | 0.123 | 0.017 |
| **17** | 3-MeC_29_ | 0.114 | **-0.395** |
| **18** | C_30_ | 0.038 | -0.008 |
| **19** | C_31_ | 0.013 | -0.167 |
| **20** | 15-MeC_31_ | -0.028 | 0.109 |
| **21** | 3,19_,_ 3,17-diMeC_31_ | 0.101 | -0.082 |
| **22** | 3-MeC_31_ | 0.063 | **-0.328** |
| **23** | 4,12-diMeC_31_ | 0.164 | -0.086 |
| **24** | 11-MeC_33_ | -0.109 | -0.068 |
| **25** | 15,17-diMeC_33_ | -0.038 | 0.021 |
